# Product authenticity versus globalisation—The Tulsi case

**DOI:** 10.1371/journal.pone.0207763

**Published:** 2018-11-26

**Authors:** Gabriele Jürges, Vaidurya Sahi, Daniela Rios Rodriguez, Eike Reich, Sukvinder Bhamra, Caroline Howard, Adrian Slater, Peter Nick

**Affiliations:** 1 Molecular Cell Biology, Botanical Institute, Karlsruhe Institute of Technology, Germany; 2 CAMAG Laboratories, Muttenz, Switzerland; 3 Biomolecular Technology Group, School of Allied Health Sciences, The Gateway, De Montfort University, Leicester, United Kingdom; 4 University of Kent, Medway School of Pharmacy, Chatham, Kent, United Kingdom; 5 BP-NIBSC Herbal Laboratory, National Institute for Biological Standards and Control (NIBSC), Blanche Lane, South Mimms, Potters Bar, Hertfordshire, United Kingdom; Aristotle University of Thessaloniki, GREECE

## Abstract

Using the Indian medicinal plant Tulsi (Holy Basil) as a case study, we have tested to what extent the discrepancy between vernacular and scientific nomenclature can be resolved, whether the presumed chemical diversity underlying the medicinal use of Tulsi has a genetic component, and whether it is possible to detect this genetic component using genetic barcoding markers. Based on four plastidic markers, we can define several haplotypes within *Ocimum* that are consistent across these markers. Haplotype II is congruent with *O*. *tenuiflorum*, while haplotype I extends over several members of the genus and cannot be resolved into genetically separate subclades. The vernacular subdivision of Tulsi into three types (Rama, Krishna, Vana) can only be partially linked with genetic differences–whereby Rama and Krishna Tulsi can be assigned to *O*. *tenuiflorum*, while Vana Tulsi belongs to haplotype I. This genetic difference is mirrored by differences in the profiles of secondary compounds. While developmental state and light quality modulate the amplitude to which the chemical profile is expressed, the profile itself seems to be linked with genetic differences. We finally develop an authentication assay that makes use of a characteristic single nucleotide polymorphism in one of the barcoding markers, establishing a differential restriction pattern that can be used to discriminate Vana Tulsi.

## Introduction

Medicinal use of plants has accompanied human civilisation over millennia. Observations in wild chimpanzees that make use of specific herbs for healing and transmit this habit by individual education [1] suggest that phytotherapy has been a central factor during the cultural evolution of humans from its very beginning. Based on the importance of plants for human culture, it is easy to understand that traditional nomenclature is predominantly shaped by human usage of plants. This functional context of vernacular plant names is often incompatible with scientific taxonomy that attempts to reflect (more or less successfully) phylogenetic relationship. This conflict had been exemplarily investigated using the case of the Tzeltal, an ethnic community of native Americans in Mexico [2] leading to the noteworthy conclusion that folk names tell “*…nothing about the structure of nature itself*, *but a great deal about our own view of this structure*.”

The discrepancy between traditional and scientific nomenclature often goes unnoticed, so long as a given plant remains in its traditional functional context. However, in the very moment when this plant is shifted into a novel functional context, these discrepancies become highly problematic for quality control and consumer protection in the importing countries.

Three examples may suffice to illustrate this point: The traditional Mesoamerican oil crop ‘Chia’ is currently booming as so called “super food” in Europe and is defined as *Salvia hispanica* L. by the EU Novel Food Regulation [3]. However, in their region of origin, the name ‘Chia’ (in the languages of the native Mesoamericans meaning just “oily”) is used for six different plants; some of them, such as *Amaranthus caudatus*, belong to completely different plant families [4]. Ambiguities of vernacular names can also lead to drastic misdeclaration of commercial products as found recently for Bamboo tea (designated as *Dan Zhu Ye* in Traditional Chinese Medicine, TCM). Many samples did not contain any Bamboo, but rather the leaves of a Chinese species of carnation, which in China is also known as “Stone Bamboo” (*Shi Zhu*) and should not be consumed by pregnant women because it activates uterine contractions [5]. So far, the most serious consequence of misunderstood vernacular taxonomy is the case of *han fangchi* (*Aristolochia fangchi*) which had been traded to Europe as (guan) fangchi (*Stephania tetrandra*), with the consequence that more than 100 women in Belgium lost their kidneys as a result of consuming a TCM based slimming therapy [6].

It is evident that problems caused by the ambiguities of vernacular taxonomy will be accentuated progressively as a consequence of economic globalisation. Already in the 1990s, the international trade volume for pharmaceutical plants was estimated to exceed 400 000 tons per year [7], a number which will have increased in the years since and does not consider the extensive proportion of medicinal plants that are directly consumed in their country of origin. Consumer safety is further deteriorated by deliberate replacement of medicinal plants by cheaper, but often inactive, substitutes–a problem that in combination with a rapidly growing internet trade, has become a major concern for a substantial proportion of commercially traded medicinal plants [8]. The mandate for consumer safety requires that vernacular names are properly assigned to scientific taxonomy. However, despite useful endeavours, e.g. the Kew Garden website for Medicinal Plant Names (http://mpns.kew.org), a Babel Fish [9] for medicinal plant nomenclature is still to be invented. This metaphor for a translation device illustrates the problem: there is no bidirectional relation in the “translation” of vernacular into scientific nomenclature, since the vernacular nomenclature is context dependent.

If the commercial declaration of traded medicinal plants must be treated with caution for the reasons mentioned above, we need methods to monitor the authenticity of traded materials. These methods should be fast, easy, and reliable. The traditional authentication of processed plant samples by microscopic diagnostics requires considerable experience and skill, and is often limited by processing, which destroys the morphological features required for determination. Chemical analysis of characteristic secondary compounds, for instance by thin layer chromatography, is frequently used for authentication, but can be misleading when the sample has deteriorated during storage or transport, or when the diagnostic compound had not accumulated in consequence of environmental fluctuations during cultivation. During the last decade, polymorphisms in specific DNA sequences, so called genetic barcodes, have attracted considerable attention, because they allow authentication of organisms based on their genotype, and, therefore, independently from tissue, environment, or developmental state.

The international Consortium for a Barcode of Life (CBOL) has investigated different marker sequences for their suitability as barcodes for different organisms. In addition to nuclear markers, such as the internal transcribed spacers of the *rRNA* genes, sequences from organelle genomes are used because they are present in multiple copies and are therefore abundant in total DNA extracted from plant material. Since mitochondrial barcodes, which are prevalent in animal molecular taxonomy, show low substitution rates in plants, barcoding of land plants frequently employs plastid markers. These markers were systematically tested with respect to different criteria, such as universality, variability between different taxa, amenability to sequencing, and the presence of sequence motifs that are conservative enough to allow the design of universal primers, but on the other hand flank sequence regions that are variable enough to be informative [10]. From this study, two regions in coding genes (*1*,*5-ribulose bisphosphate carboxylase Large subunit*, *rbcL; maturase K*, *matK*), and one intergenic spacer (*trnH-psbA IGS*) were found to be the most powerful single-locus barcodes. However, compared to the discriminative power of animal barcodes, the resolution of these plant barcodes remains limited–while the resolution is sufficient to discern genera, these barcodes are often not able to discriminate down to the species level, because the variation within a species often exceeds the taxon gap between species [11].

A model case for the conflict of vernacular and scientific nomenclature is represented by the Basils (genus *Ocimum*). From molecular phylogeny, the origin of the genus can be inferred to be tropical Asia [12] with the centre of diversity in the Indian subcontinent. The Basils are probably the medicinal plants with the longest documented tradition spanning around four millennia with first records dating to the Vedic scripts. As might be expected from such a long and intricate history of human use, the traditional nomenclature of the Basils (Tulsi, Tulasi) is complex, ambiguous, and, in the last consequence, not projectable on to any scientific taxonomy. On the other hand, the trading volume is, with around 100 t of essential oils per year, quite considerable [13]. Depending on their appearance and use, the Tulsis are differentiated in a kind of binomial nomenclature–for instance, the dominant form, called Rama Tulsi, is used for religious purposes and usually “translated” as *O*. *tenuiflorum* L. [14]. The purple Krishna Tulsi, named after its resemblance to the colour of the Hindu deity Krishna, is preferred for medical use and also assigned to *O*. *tenuiflorum* L. [14, 15]. In contrast, Rama Tulsi (synonymous with Sri Tulsi), probably due to its importance for worship, is sometimes assigned to (the now outdated) *O*. *sanctum L*. [16]. A third type, Vana Tulsi (the meaning “forest” Tulsi may indicate that it is often collected in the wild), is mostly “translated” as *O*. *gratissimum* L. [14, 16] although it is also used as a name for *O*. *canum* (an outdated name for *O*. *americanum* L., [17]). There is a whole cosmos of additional types of Tulsi that partially stand for other species of *Ocimum*, such as Babui Tulsi for *O*. *basilicum* L. [15] that can also appear as Kali Tulsi [18], while Kala Tulsi stands for *O*. *americanum*, but in the same source [18] is also used as a synonym for Sri Tulsi, while Ram Tulsi is also used for *O*. *gratissimum* L. Only a few species, such as *O*. *kilimandscharicum*, which was brought to India from East Africa, seem to carry a traditional name (Kapur Tulsi from the Hindi word for camphor) that is unequivocal. The *tenuiflorum* type of Tulsi might even show further differentiations, because for certain treatments, such as the expelling of worms or wound healing, a form of Tulsi is prescribed that is called Surasaadi [15].

Now, this highly ambiguous traditional nomenclature hits a global market, where Tulsi is praised as an “adaptogen” for the stressed Westerner [19] and traded in continuously increasing amounts. This conflict of nomenclature will inevitably culminate in serious problems for quality control and consumer information. The situation is complicated further by the fact that several chemovars exist that strongly differ in their composition of essential oils–for instance, commercial use discriminates between a methyl-cinnamate, a camphor, and a citral chemotype of *O*. *americanum*, while for *O*. *basilicum* four different chemotypes are described [18]. However, it is not clear, to what extent these chemical differences are determined by genetics (“nature”), and to what extent by differences in the environment (“nurture”).

We therefore addressed the question, whether chemical diversity (underlying the medical effect of Tulsi), and genetic diversity (as detectable by genetic barcodes) can be assigned, and to what extent the traditional differentiation of Tulsi into Rama, Krishna, and Vana Tulsi correlate to genetic differences that can be used for molecular authentication in commercial samples exported from India.

## Materials and methods

### Plant material, cultivation, and light conditions

The study was built around a collection of *Ocimum* established in the Botanical Garden of the Karlsruhe Institute of Technology, which had been raised to the flowering stage and identified based on taxonomical keys [20–22]. For the subtypes of Tulsi, seeds from plants that have been validated by trained Ayurvedic herbalists (Pukka, Bristol, UK) were used. To avoid ambiguities caused by the numerous synonyms for *Ocimum* species, only the accepted names given in the Botanic Garden, Kew Plant List (http://www.theplantlist.org/) are used throughout. Internal accession ID and source, identity after morphological authentication, and Genbank accession numbers of the sequences isolated from these accessions are given in **Table 1**. Two commercial samples were also included in the study. Sample 1 was declared as a mixture between Rama Tulsi, Krishna Tulsi, and Vana Tulsi, while sample 2 was declared as a mixture between Rama Tulsi (*O*. *sanctum*), Krishna Tulsi (*O*. *sanctum*), Vana Tulsi (*O*. *gratissimum*) and cinnamon, ginger, cardamom, and black pepper. For the analysis, sample 1 was used directly, while for sample 2, the non-foliar components were sorted out. Plants were cultivated in individual pots, on a commercial white peat clay substrate (Floraton 3, Floragard, Oldenburg, Germany). All genotypes were raised in parallel in the greenhouse at a temperature of 22°C ± 5°C during daytime, and 18° ± 5°C during the night. The light period was 16 h ± 2 h at around 108 μM^.^sec^-1.^m^-2^ ± 30 μM^.^sec^-1.^m^-2^ of photosynthetically available radiation at the Botanical Garden of the Karlsruhe Institute of Technology (49.008681 N, 8.403886 W). For the light quality experiments, the plants were raised in custom-made growth cabinets (Cerovac, Bretten, Germany) equipped with either white light, or white light supplemented with red and blue LEDs (655 nm, 447 nm, Luxeon PLR700-5-RB-PWM), with UV-A (365 nm, Nichia STS-DA1 2394 G) or with UV-B (310 nm, Tianhui-UV310-5050C-A) adjusted to a photosynthetically available radiation of 165 μM^.^sec^-1.^m^-2^ ± 30 μM^.^sec^-1.^m^-2^ and continuous illumination.

**Table 1:** *Ocimum* accessions used in this study; Internal accession ID and source, identity after morphological authentication, and Genbank accession numbers of the sequences isolated from these accessions. Note: under accession the designation of the provider is listed, in case that not an accepted name has been used, the official name, based on The Plant List (http://www.theplantlist.org/) has been added in brackets.

Taxon	Identity	ID	Source	rbcL	matK	trnL-F igs	trnH-psbA igs
*O*. *basilicum* L.	*O*. *basilicum* L.	5192	Rühlemanns	MF326408	MF379667		MF784535
*O*. *basilicum* L. var. *thyrsiflorum*	*O*. *basilicum* L.	7562	Rühlemanns	MF326419	MF379671		
*O*. *citriodorum*	*O*. *x africanum* Lour.	5748	Rühlemanns	MF326409	MF379668		MF784537
*O*. *americanum* var. *pilosum*	*O*. x *africanum* Lour.	7537	Rühlemanns	MF326418	MF379670		MF784538
*O*. *kilimandscharicum*	*O*. *kilimandscharicum* Gürke	7809	BG Würzburg	MF326421	MF379673		
*O*. *kilimandscharicum*	*O*. *kilimandscharicum* Gürke	7810	BG Würzburg	MF326422	MF379674		MF784539
*O*. *americanum*	*O*. *americanum* L.	7811	BG Greifswald	MF326423	MF379675		MF784536
*O*. *tenuiflorum*	*O*. *tenuiflorum* L.	5751	Rühlemanns	MF326411	MF399413	MF447881	MF784540
*O*. *tenuiflorum*	*O*. *tenuiflorum* L.	8097	CAMAG	MF326412	MF379676	MF447880	MF784541
*O*. *tenuiflorum*	*O*. *tenuiflorum* L.	8098	CAMAG	MF326413	MF379677	MF447879	MF784542
*O*. *tenuiflorum*	*O*. *tenuiflorum* L.	8099	CAMAG	MF326414	MF379678	MF447878	
*Rama Tulsi*	*O*. *tenuiflorum* L.	8256	Pukka	MF326415	MF379679	MF447876	MF784543
*Krishna Tulsi*	*O*. *tenuiflorum* L.	8257	Pukka	MF326416	MF379680	MF447877	MF784544
*Vana Tulsi*	*O*. *americanum* L. ?	8258	Pukka	MF326417	MF379681	MF447875	MF784545
*O*. *carnosum*	*O*. *campechianum* Mill.	7564	Rühlemanns	MF326420	MF379672		MF784557
*O*. *gratissimum*	*O*. *gratissimum* L.	5749	BG Bonn	MF326410	MF379669		MF784560

### DNA extraction, PCR, oligonucleotide primers, and sequence analysis

A small amount (50 mg) of leaf material was harvested, immediately shock-frozen in liquid nitrogen, and stored at -80°C. The frozen material was ground using a high-throughput disruptor (TissueLyser, Qiagen, Germany), and the DNA extracted using the InnuPrep Plant DNA Kit (Macherey-Nagel) following the instructions of the producer. Quality was controlled spectrophotometrically (Nano-Drop 1000, peQLab) and by gel electrophoresis. The genomic DNA (50 ng template) was then subjected to a PCR using primers for different plastidic barcoding markers (details are given in **Table 2**) with an initial denaturation at 95°C for 2 min, followed by 35 cycles of 30 sec denaturation at 95°C, 30 sec annealing at 60°C, and 60 sec synthesis at 68°C using a conventional Taq polymerase (New England Biolabs, Frankfurt). Final elongation was 5 min at 68°C. Amplicons were then separated by 1.5% agarose gel electrophoresis at constant voltage of 100 V, results were documented by a digital gel documentation system using the fluorescence of SYBRSafe as label. Amplicons were sent for sequencing (GATC, Konstanz, Germany) from both sides, the two sequence reads were aligned using the Alignment Explorer of MEGA phylogenetic software (www.megasoftware.net) and checked for quality by visual inspection of the electropherograms, and by a BLAST search to validate sequence identity. Homologous sequences for Ocimum and two outgroups (*Hanceola sinensis*, *Nepeta cataria*) were retrieved from GenBank and aligned after filtering for identity (sequences assigned to the genus, but not a species, were not considered, because they are not informative for this purpose) and authenticity (only sequences from taxa that had been determined by the submitters were considered, in case of doubt, this point was cross-checked by corresponding with the submitting authors). The alignments were trimmed and then used to construct phylogenetic trees either based on the Neighbour-Joining algorithm (model: maximum composite likelihood with uniform rates) or the Maximum Likelihood (model: Tamura-Nei with uniform rates, using Nearest Neighbour Interchange as heuristic algorithm to infer the tree), and bootstraps derived from 1000 replicates, again making use of the MEGA phylogenetic software. In addition to the individual loci, a concatenated alignment was used to infer the phylogeny.

**Table 2:** Oligonucleotide primers used in the current study. Amplicon lengths are predicted for different Ocimum taxa investigated in the current study.

target	Sequence	Amplicon (bp)
rbcL	F: 5-ATGTCACCACAAACAGAGACTAAAGC-3 R: 5-CGTGGTGGACTTGATTTTAC-3	599
matK	F: 5-ACCCAGTCCATCTGGAAATCTTGGTTC-3 R: 5-CGTACAGTACTTTTGTGTTTACGAG-3	878
trnL-F igs	F: 5-CGAAATCGGTAGACGCTACG-3 R: 5-ATTTGAACTGGTGACAGAG-3	803–1209
trnH-psbA igs	F: 5-GTTATGCATGAACGTAATGCTC-3 R: 5-CGCGCATGGTGGATTCACAATCC-3	420–446

### Light microscopy

To determine the density of glandular scales, ten leaves per accession and light condition were analysed. Only leaves without any indication of yellowing or wilting were considered. Glandular scales were scored at five fixed positions of the adaxial and abaxial face of each freshly excised leaf (viewed as wholemount specimens) in a window of 430 μm x 330 μm at 20 x magnification under a bright-field microscope (M420, Leica, Bensheim, Germany) with a digital image acquisition system (DFC 500, Leica, Bensheim, Germany). To detect genetic differences between the accessions, the scale numbers per scoring window were compared in relative units between plants raised side by side under identical lighting conditions. Results were analysed using a one-factorial ANOVA for homogeneity of variance, before the detected differences were tested pairwise for significance using a two-sampled t-test.

### Chemical profiling of flavonols and essential oils by HP-TLC

Dried leaf material was powdered (Tube Mill, IKA) and 0.5 g of powdered specimen were ultrasonicated for 10 min with 5 mL of methanol (Ultrasonic Bath SW 3H, Sono Swiss) and briefly spun down to remove cell debris (EBA21, Hettich). Supernatants were separated using HP-TLC and analysed for the patterns of flavonoids or of essential oils, respectively. Aliquots of 10 μL for each sample were loaded as 8-mm bands on a 200 mm x 100 mm HPTLC glass plate (Si 60 F_254_, Merck, Darmstadt, Germany) using an Automatic TLC Sampler (ATS4). After development in an Automatic Development Chamber (ADC2) under standardised conditions (20 min chamber saturation, 33% humidity with magnesium chloride, development distance of 70 mm from lower edge, drying time 5 min), the plates were derivatised by dipping (5 cm/s, dwell time 0 s, Immersion Device) and documented using the Visualizer. The HPTLC equipment was made by CAMAG, Muttenz Switzerland, and the *vision*CATS software 2.3 (CAMAG) was used for instrument control and data acquisition. Choice of standards, as well as developing solvents and derivatisation reagents were dependent on the respective group of compounds. For the essential oils standard solutions of eugenol (0.5 mg/mL), methyleugenol (0.5 mg/mL), and ursolic acid (0.25 mg/mL) in methanol were used as System Suitability Test (SST) and toluene/ethyl acetate (85/15) as developing solvent. The plates were derivatised with 0.5% of p-anisaldehyde in acetic acid/sulfuric acid/methanol (10/20/170)) and then heated (TLC Plate Heater III) at 100°C for 3 minutes. For flavonoids, standard solutions of rutin (0.5 mg/mL), hyperoside (0.125 mg/mL), and rosmarinic acid (0.5 mg/mL) in methanol were used as SST and development was conducted in ethyl acetate/water/formic acid (15/1/1). For derivatisation, the plates were preheated for 3 min at 100°C and then derivatised with natural product reagent (0.5% w/v of 2-aminoethyl diphenylborinate in ethyl acetate) and subsequently with PEG reagent (5% w/v of polyethylene glycol in dichloromethane). The results were visualised under UV366 nm (flavonoids) or under white light (essential oils).

### Assay to discriminate ‘Tulsi’ types in commercial samples

For commercial samples, the procedure had to be adapted. Here, the frozen material was ground with liquid nitrogen using a mortar and pestle, and the DNA extracted using the Invisorb MSpin Plant Mini Kit (Stratec Molecular) following the instructions of the producer. Further, for the PCR 10 mg/ml of bovine serum albumin were added. Amplicons were digested by the restriction enzyme HinfI (New England Biolabs, Frankfurt). For the reaction, 10 μl of the PCR product was used and digested following the instructions of the producer. The products were then separated by 2% agarose gel electrophoresis at constant voltage of 100 V and results were documented by a digital gel documentation system using the fluorescence of SYBRSafe as label.

## Results

### Flower morphology of different Basils varies in geometry

**Fig 1:**
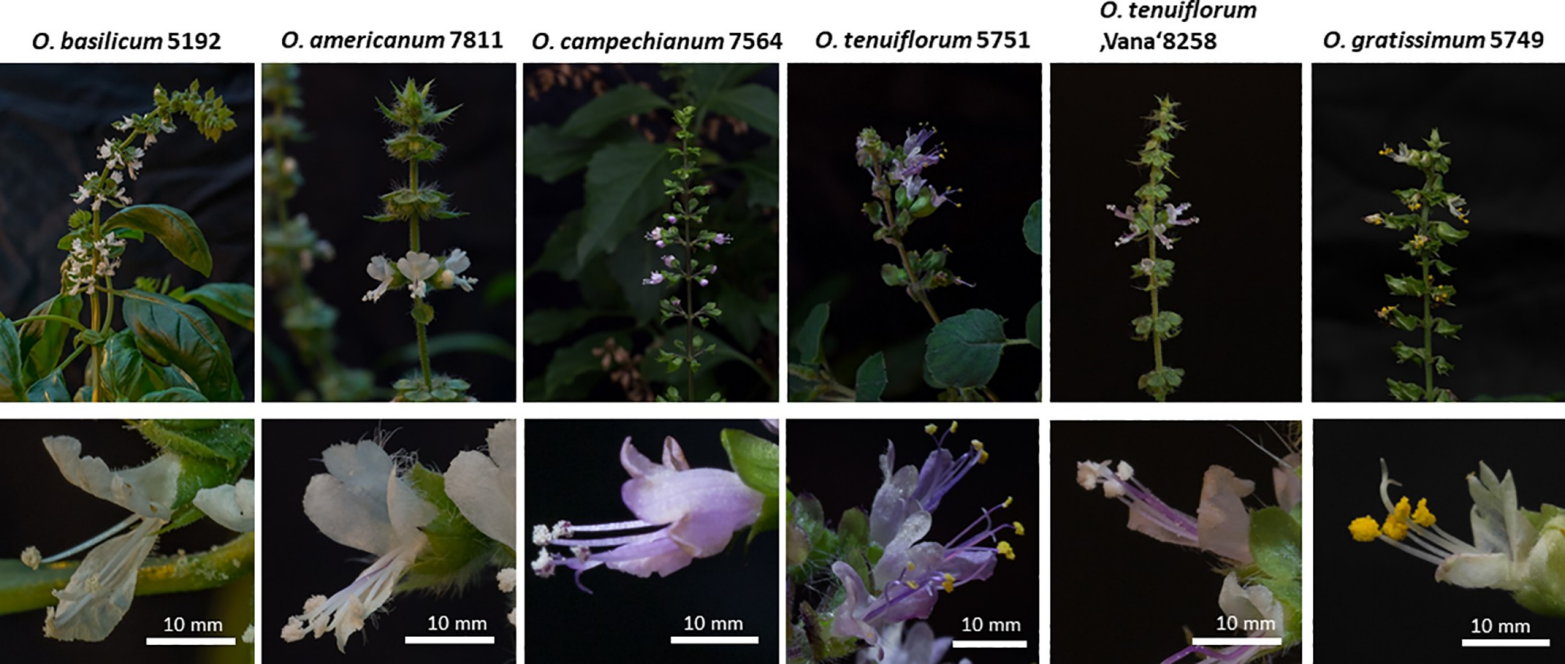
Representative specimens of *Ocimum* accessions used in the current study in the flowering stage. The upper row shows the inflorescence, the lower row shows close-ups of individual flowers with details of morphology. Size bar 10 mm.

Due to the pronounced morphological variability of vegetative organs, the determination of *Ocimum* species is far from trivial. All accessions were therefore raised to the flowering stage and then their identity verified (and sometimes updated) based on floral traits. Here, a considerable variation in the geometry of flower organs can be observed: while *O*. *basilicum* and *O*. *americanum* possess a well-developed upper lip (**Fig 1**), this organ is shorter in *O*. *kampechianum*, *O*. *tenuiflorum*, *O*. *gratissimum*, as well as in the validated specimen of ‘Vana Tulsi’ in our sample. The lower lip was long in *O*. *basilicum*, slightly retracted in *O*. *americanum* and *O*. *campechianum*, shorter in both *O*. *tenuiflorum* and in ‘Vana Tulsi’, and very short in *O*. *gratissimum*. As a result, the sexual organs in *O*. *basilicum* were contingent with the corolla, while they protruded slightly in *O*. *americanum*, *O*. *campechanium*, and in ‘Vana Tulsi’. In *O*. *tenuiflorum* and, even more pronounced in *O*. *gratissimum*, the anthers were almost freely exposed around 10 mm beyond the edge of the lower lip. These differences in geometrical relations are likely to influence pollination mechanisms or possibly even the spectrum of pollinators, leading to the question of whether they might be linked with genetic channelling leading to incipient speciation. Mutations in barcoding markers, although not under selective pressure, should then segregate between the different lineages.

### The conservative barcoding marker *rbcL* supports a distinct *tenuiflorum* clade

**Fig 2:**
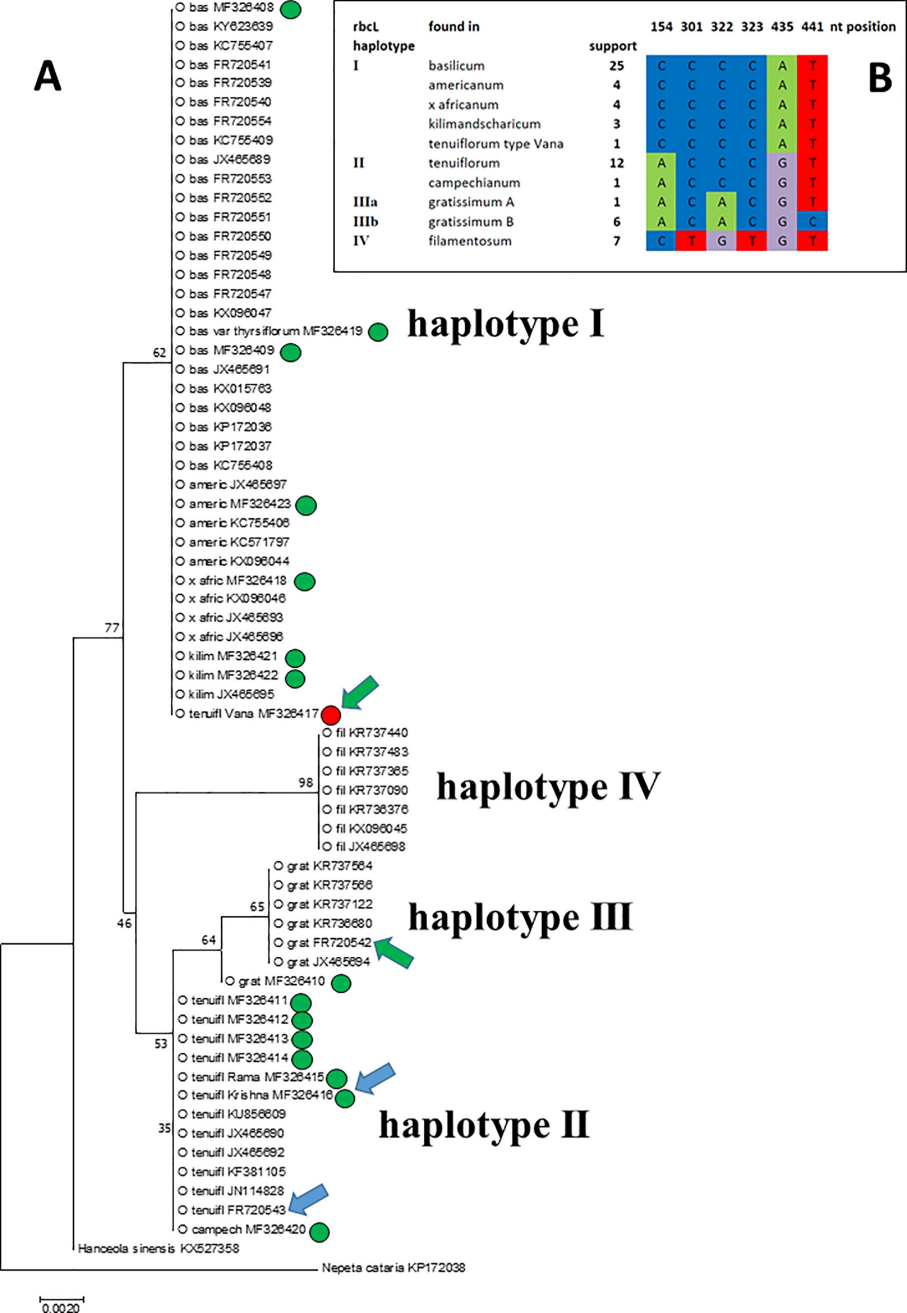
Molecular relationships inferred from the RubisCo large subunit (*rbcL*) marker. **A** Evolutionary relationship of 65 sequences for the *rbcL* marker from *Ocimum*, along with one sequence from *Nepeta cataria* and *Hanceola sinensis*, respectively, used as outgroups inferred using the Neighbour-Joining algorithm. Bootstrap values are derived from 1000 replicates. O bas *O*. *basilicum*, O americ *O*. *americanum*, O x afric *O*. x *africanum*, O kilim *O*. *kilimandscharicum*, O tenuifl *O*. *tenuiflorum*, O campech *O*. *campechianum*, O fil *O*. *filamentosum*, O grat *O*. *gratissimum*. GenBank accession numbers are shown with each accession, circles represent sequences that had been isolated in the course of the current study, red circle represents the sequence for ‘Vana Tulsi’ that clusters outside of *O*. *tenuiflorum*. Blue arrows indicate two accessions that have been reported as ‘Krishna Tulsi’ (both as *O*. *tenuiflorum*), green arrows indicate two accessions that have been reported as ‘Vana Tulsi’. Roman numbers represent four main haplotypes. **B** Signatures of the four main haplotypes defined by the *rbcL* marker, numbers indicate the nucleotide position in the alignment (**S1 Appendix**). Support is defined as the number of sequences deposited in GenBank that show this signature. Note that the sequence of ‘Vana Tulsi’ falls into cluster I comprising *O*. *basilicum*, *O*. *americanum*, *O*. x *africanum*, and *O*. *kilimandscharicum*.

Among the common barcoding markers, the 1,5-ribulose bisphosphate carboxylase Large subunit (*rbcL*) is considered to be one of the most conserved loci, because mutations in the coding sequence can impair carbon fixation in the dark reaction of photosynthesis. Therefore, the *rbcL* marker, while very useful for the discrimination of families and genera, usually shows only poor resolution below the family level [23]. When we constructed an alignment of sequences from our validated specimens along with reference sequences recovered from GenBank (**S1 Appendix**) and inferred a phylogenetic tree using the Neighbour Joining algorithm, we found that 6 of the 451 positions included were parsimony informative and allowed delineation of four haplotypes (**Fig 2A**). Haplotype I included all accessions of *O*. *basilicum* (25 sequences), but also *O*. *americanum* (4 sequences), *O*. *x africanum* (4 sequences), *O*. *kilimandscharicum* (3 sequences), as well as our validated ‘Vana Tulsi’ (**Fig 2A**, green arrow and red circle). All accessions reported as *O*. *tenuiflorum* (12 sequences) as well as our validated *O*. *campechianum* sample formed a clearly delineated second cluster. This also included our validated ‘Rama Tulsi’ and ‘Krishna Tulsi’ (blue arrow) accessions, as well as one other GenBank sequence that had been deposited as ‘Krisha Tulsi’ (blue arrow), which should probably read ‘Krishna Tulsi’. A second sequence deposited in GenBank as ‘Vana Tulsi’ (green arrow) belongs to the *O*. *gratissimum* clade and was declared as such. Both sequences come from a comparative study on Lamiaceae in commercial samples and derive from fresh material collected in the Botanical Garden of Milan without any mention of morphological verification [24]. A third clade comprises 7 sequences from *O*. *gratissimum* that neighbour *tenuiflorum* and are relatively distant from the *basilicum* haplotype. A fourth, more distant, clade comprises 7 sequences that were available for *O*. *filamentosum*. To test the validity of this topology, we have reconstructed the tree using the Maximum Likelihood algorithm. Although most clades were not statistically supported, the two trees showed the same topology (**S1 Fig**).

### The distinct *tenuiflorum* clade is supported by other plastidic markers

Since the *rbcL* marker is very conservative, we tested other plastidic barcoding markers with higher rates of substitution. This should allow validation of the assignment of ‘Vana Tulsi’ to haplotype I (comprising *O*. *basilicum*, *O*. *americanum*, *O*. x *africanum*, and *O*. *kilimandscharicum*) and to see, whether below the four clades, defined by the *rbcL* marker, further subclades could be resolved. When we aligned our sequences for *matK* with sequences recovered from GenBank (**S2 Appendix**), we could see that this marker, indeed, produced a much higher number (30 in an alignment of 720) of informative positions (**S2 Fig**). In addition, within the 35 sequences of haplotype I, two slightly different subclades emerged, although these could not be assigned to one of the four species (*O*. *basilicum*, *O*. *americanum*, *O*. x *africanum*, and *O*. *kilimandscharicum*) in the clade. Likewise, the above mentioned doubtful ‘Krishna Tulsi’ differed in 2 of the 30 positions from the other 16 sequences for *tenuiflorum*, and *O*. *campechianum* along with *O*. *carnosum* diverged slightly from *tenuiflorum*. The *O*. *gratissimum* clade remained separate from *O*. *tenuiflorum* (while being subdivided into two separate branches, a tendency already seen in the *rbcL* analysis), and also *O*. *filamentosum* remained delineated. In summary, while the *matK* marker was able to resolve further subdivisions, it confirmed the four major clades seen with the *rbcL* marker as well as the position of ‘Vana Tulsi’ within haplotype I, and outside of *O*. *tenuiflorum*. Both conclusions remained valid, when two additional plastidic barcodes (*trnL-F* intergenic spacer, *trnH-psbA* intergenic spacer) were aligned, although the alignment of these intergenic spacers were, as to be expected, generally more variable and even contained gaps (S3 and S4 **Appendices**). The two subclades within *gratissimum* that were previously observed with *rbcL* and, more clearly, with *matK*, became extremely distinct on the basis of both the *trnL-F* intergenic spacer (**S3 Fig**) and the *trnH-psbA* intergenic spacer (**S4 Fig**). Also, haplotype I was further differentiated into subgroups (although with relatively low bootstrap support and not assignable to a specific species). However, for both markers, the numerous sequences for *O*. *tenuiflorum* remained as a separate, monophyletic group. Likewise, ‘Vana Tulsi’ although grouping with haplotype I, remained separate from the remaining members of this clade. Only in one of many polymorphisms in the *trnL-F* marker was ‘Vana Tulsi’ seen to group with *O*. *tenuiflorum* and not with *O*. *basilicum* (**S3B Fig**, green square). Otherwise, ‘Vana Tulsi’ clustered with a subgroup of haplotype I harbouring sequences from *O*. *basilicum*, *O*. *americanum*, *O*. x *africanum*, and *O*. *kilimandscharicum*. In contrast, ‘Krishna Tulsi’ and ‘Rama Tulsi’ belonged to *O*. *tenuiflorum*, no matter which barcoding marker was considered.

**Fig 3:**
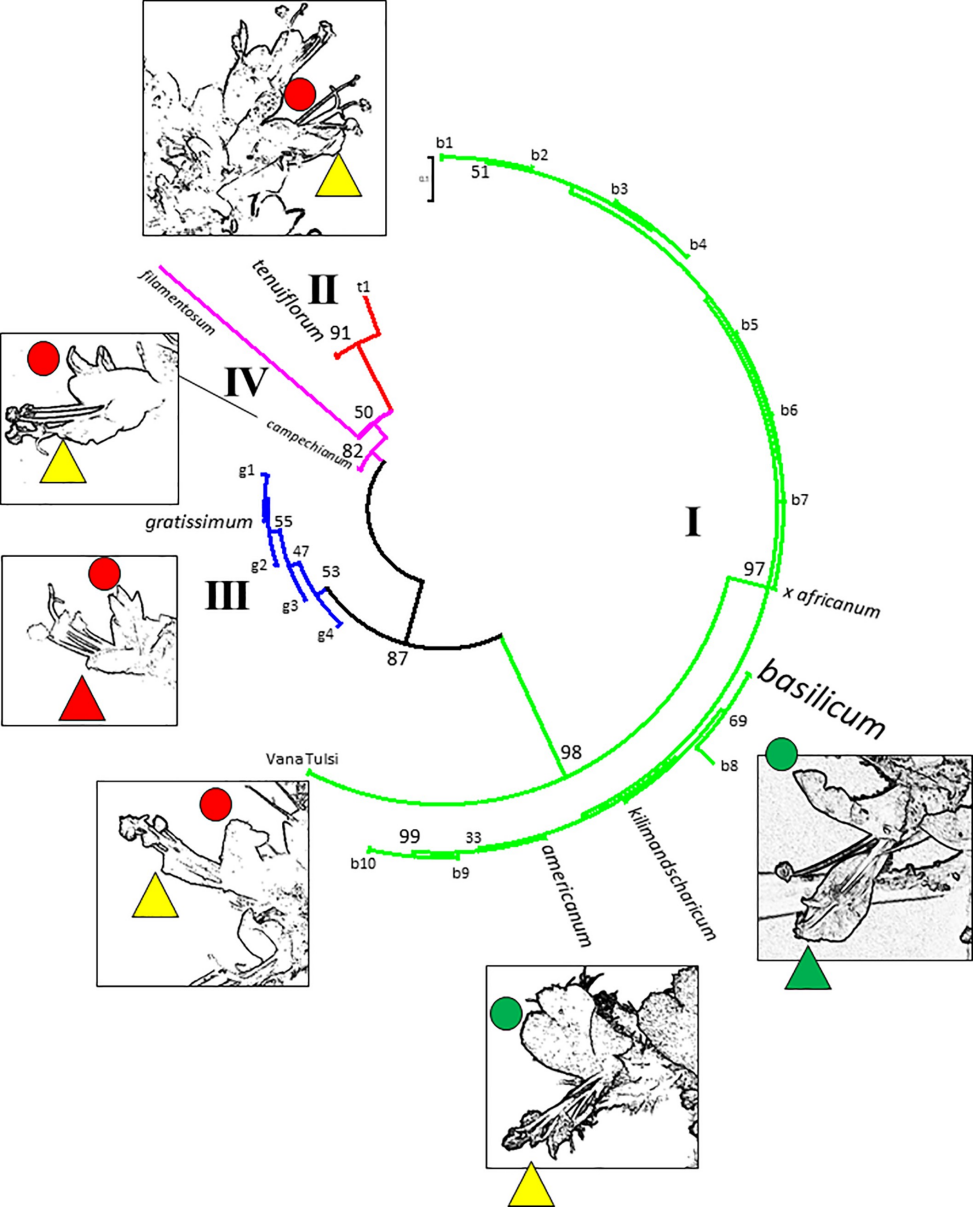
Molecular relationships inferred from the informative positions in a composite alignment of *rbcL*, *matK*, trnF-L intergenic spacer, and the *trnH-psbA* intergenic spacer based on the 79 informative positions in this composite alignment. The inset shows the flower morphology of the different clades. The colour code for upper (circles) and lower (triangles) lips is green long, yellow intermediate, red short. Haplotypes that deviate from the consensus for the respective clade (b *basilicum*/*americanum*/*x africanum*/*kilimandscharicum* clade, g *gratissimum* clade, t *tenuiflorum* clade) are indicated by numbers).

When the four markers were concatenated into a combined matrix and the informative positions used to construct a phylogenetic tree, the relationships emerged very clearly and with high bootstrap support (**Fig 3**): *O*. *basilicum*, *O*. *americanum*, *O*. x *africanum*, and *O*. *kilimandscharicum* formed one common clade (97%), ‘Vana Tulsi’, although slightly (but significantly) different, was clearly located in this clade, but significantly (98%) separated from the other accessions. In contrast, *O*. *tenuiflorum* formed a well supported (91%) monophyletic group with considerable distance from the *basilicum* clade. The only (small) deviation in this block was the ‘Krishna Tulsi’ accession from the Botanical Garden in Milan mentioned above (t1). *O*. *filamentosum* and *O*. *campechianum* formed sister groups that appeared to be monophyletic and were delineated from *O*. *tenuiflorum*, but at lower bootstrap values (50% in case of *O*. *filamentosum*, 82% in case of *O*. *campechianum*) In contrast, *O*. *gratissimum* which was also formed a well supported clade (87%) was split into subgroupsthat were more differentiated than the subgroups in the *basilicum* clade, although showing low bootstrap values (~50%). When flower morphology was projected onto this tree, the *basilicum* clade differed from the other clades due to its longer corolla, while in the *tenuiflorum*, *campechianum*, and *gratissimum* clades the corolla was short, such that the anthers protruded significantly. Interestingly, ‘Vana Tulsi’ was closer to the non-*basilicum* clades if flower morphology was considered, although with regard to the genetic markers it clearly associated with the *basilicum* clade.

### Chemotype depends on genotype, but its expression is modulated

**Fig 4:**
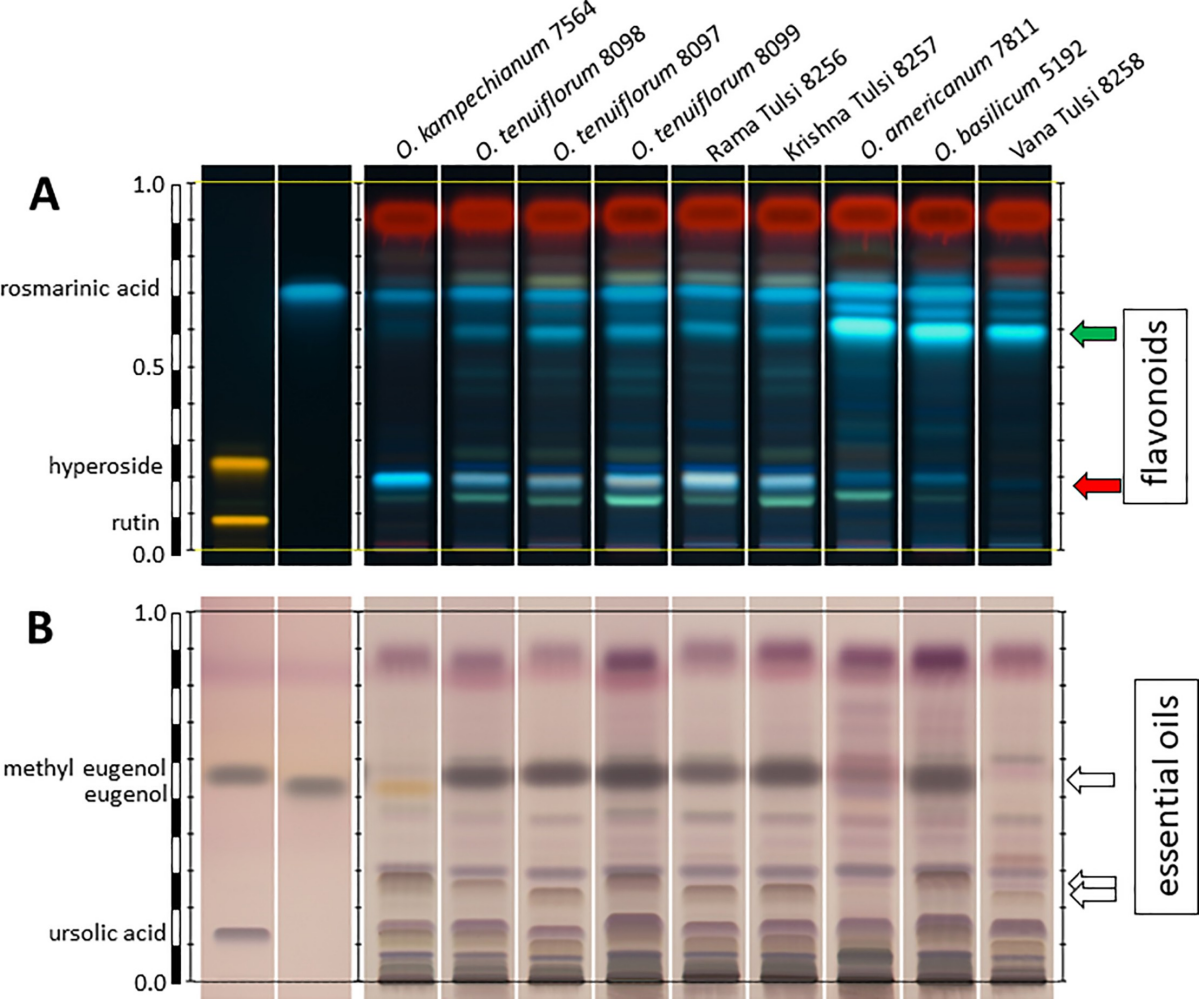
Chemical profiling of different *Ocimum* accessions raised side by side in the greenhouse under white light over six months. Flavonoids (**A**) and essential oils (**B**) were probed by HT-TLC along with reference standards (in case of flavonoids, rutin and hyperoside, in case of essential oils methyl eugenol and ursolic acid). Arrows indicate type-specific bands, green arrow indicates a prominent band in the flavonoid band that is characteristic for accessions belonging to haplotype I in Fig 3, while red arrow indicates a flavonoid band that is characteristic for accessions belonging to haplotype II and IV accessions in **Fig 3**.

To become of practical relevance for consumer protection, the genetic differences between different Tulsi types have to be reflected by chemical differences. Alternatively, the chemical differences might derive from environmental factors independent of genotype. In fact, the different chemotypes found in the Basils are traditionally interpreted as resulting from cultivation in different areas rather than having a genetic basis [18]. However, to our knowledge, this has never been addressed by systematic experimentation. We therefore raised different genotypes side by side under different light qualities and then profiled two groups of bioactive fractions by HP-TLC–the water-soluble flavonoids, and the lipophilic essential oils. In a preparatory study, where the three types of Tulsi were compared through development (**S5 Fig**), we observed that the profile of flavonoids was strongly dependent on developmental stage, whereby the abundance of compounds increased with the age of the plant. As expected, the abundance of flavonoids in commercial samples of the same accessions (that had been raised under the tropical sun of India) was higher than in plants raised in the greenhouse. However, despite these modulations, the profile of ‘Vana Tulsi’ remained clearly distinct from that seen in ‘Rama Tulsi’ or ‘Krishna Tulsi’. Thus, while the expression of the compound pattern clearly varied depending on developmental stage and cultivation conditions, the pattern itself was constant for a given genotype. In the next step, we compared flavonoid profiles (**Fig 4A**) and essential oil profiles (**Fig 4B**) for different accessions of *Ocimum*. Despite an overall congruence of these patterns, there were specific bands, where the accessions differed clearly. For instance, in the flavonoid fingerprint two blue fluorescent bands at R_f_ ~ 0.6 were characteristic for accessions belonging to haplotype I as defined in **Fig 3**. Interestingly, this band was also seen in ‘Vana Tulsi’ (**Fig 4A**). Conversely, a green fluorescent band at R_f_ ~ 0.2 was seen in accessions belonging to *O*. *tenuiflorum* (including ‘Rama Tulsi’ and ‘Krishna Tulsi’) and *O*. *campechianum*, but was weak or even absent in accessions from haplotype I as well as in ‘Vana Tulsi’. Overall, the flavonoid profile could be matched to the genetically defined haplotypes. The essential oil pattern also showed accession-dependent differences (**Fig 4B**). While the essential oil pattern of ‘Vana Tulsi’ clearly differed from the tested accessions of *O*. *tenuiflorum* including ‘Rama Tulsi’ and ‘Krishna Tulsi’, it was also distinct from *O*. *basilicum* and *O*. *americanum* (that were also variable). Generally, the profiles seen for the essential oils showed a larger variation between the genotypes and revealed chemotypic differences that were more pronounced than the genetic differences.

**Fig 5:**
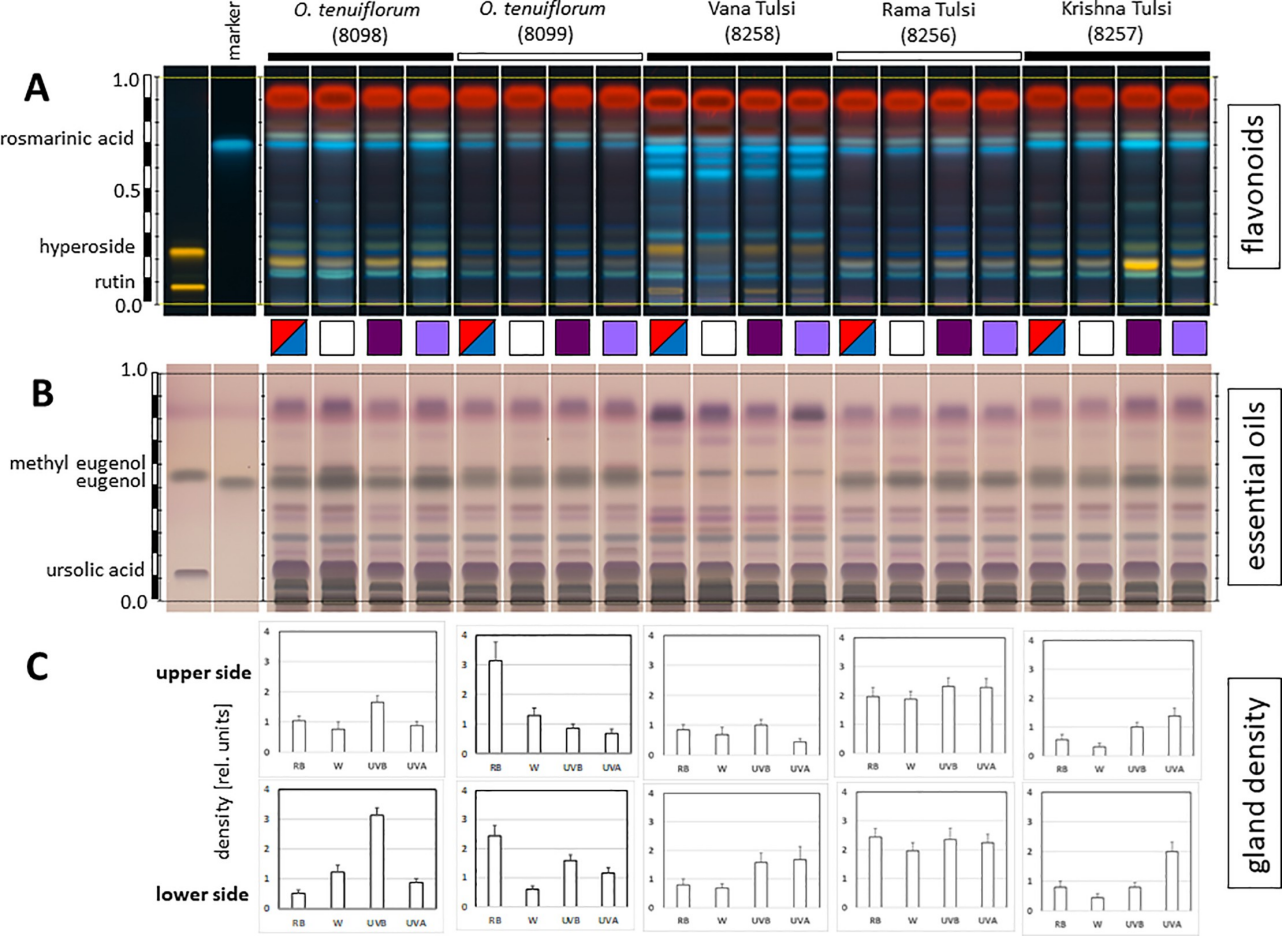
Effect of light quality on chemical profiles and glandular scale number in *Ocimum tenuiflorum*, ‘Rama Tulsi’, ‘Krishna Tulsi’, and ‘Vana Tulsi’ accessions raised side by side in the greenhouse under equal fluence rates (108 μM^.^sec^-1.^m^-2^ ± 30 μM^.^sec^-1.^m^-2^) of either white light (WL), white light supplemented with red and blue light (655 nm, 447 nm, RB), long-wavelength UV (365 nm, UVA) or short-wavelength UV (310 nm, UVB) over six months. Flavonoids (**A**) and essential oils (**B**) were probed by HT-TLC along with reference standards (in case of flavonoids, rutin and hyperoside, in case of essential oils methyl eugenol and ursolic acid). Profiles for one accession are shown side by side, indicated by colour symbols (RB, red-blue squares, WL white squares, UVB dark-violet squares, UVA light-violet squares). C mean densities of glandular scales on the upper, adaxial, and the lower, abaxial, side of fully expanded leaves given in relative units. Data represent mean values and standard errors from two independent experimental series comprising each three individual plants per accession and ten leaves per plant for each genotype.

The striking trait of ‘Krishna Tulsi’ is its purple colour caused by anthocyanins. Since the expression of anthocyanins is often under the control of photoreceptors, we investigated the extent to which differences in the chemical profiles of ‘Vana Tulsi’ versus ‘Rama Tulsi’ and ‘Krishna Tulsi’ might be caused by different lighting conditions during cultivation. This was systematically tested by raising the plants side by side under equal fluence rates of light but under light of different spectral composition. In addition to conventional white light, we tested white light complemented with red and blue light, which should not only activate chlorophyll (this was excluded by keeping the photosynthetically available radiation constant throughout all parallels of this experiment) but would also activate type-II phytochromes (red light) as well as type-I phytochromes (blue light). A third set used white light complemented with long-wavelength UV-A (365 nm) to activate cryptochromes, and a fourth set with white light complemented with short-wavelength UV-B (310 nm) should active the UVR8 receptor. While we were able to see certain modulations in the flavonoid patterns (for instance, in ‘Krishna Tulsi’ a yellow band at R_f_ ~ 0.18 became upregulated under UV-B, i.e. under conditions, where anthocyanin expression in most plants is most active), the profile of flavonoids remained unaltered (**Fig 5A**), which was also true for the pattern for essential oils (**Fig 5B**). The plants were clearly responsive to the changes in light quality, as was visible from the differences in the density of oil glands for different light qualities (**Fig 5C**). Here, the different genotypes were not only different in terms of overall ability to produce glandular scales, but also in terms of wavelength dependency and responsiveness to altered light quality. For instance, ‘Rama Tulsi’ was effectively insensitive to the modulation of wavelength, while *O*. *tenuiflorum* accession 8099 was highly responsive to red and blue light and *O*. *tenuiflorum* accession 8098 was most responsive to UV-B light. ‘Vana Tulsi’ and ‘Krishna Tulsi’ generally produced a lower number of glands but were stimulated to do so by short-waved irradiation (mostly UV-A).

In summary, although the different *Ocimum* accessions respond differently to altered light qualities, and although the amplitude of compound accumulation is modulated by light, and (in a pronounced manner) by the age of the plant, the compound profile itself seems to be quite variable. The conclusion from these experiments is that chemotype depends on genotype, but its expression is modulated by environment and development.

### Assay to discriminate ‘Tulsi’ types in commercial samples

**Fig 6:**
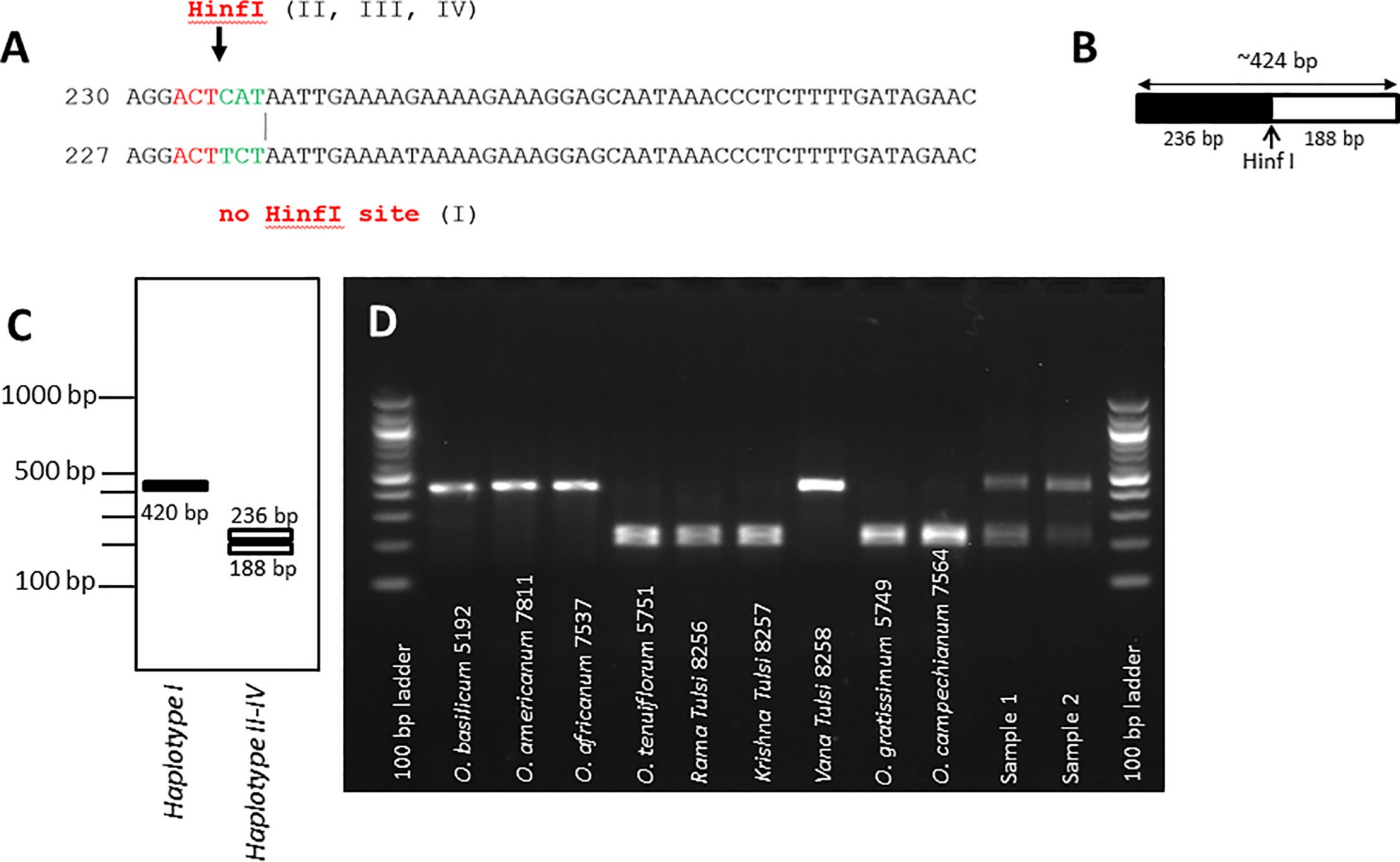
Discrimination of haplotype I and haplotypes II-IV using restriction length polymorphism of the *trnH-psbA IGS* marker. **A** Sequence polymorphism between the haplotypes leading to a differential Hinf I recognition site in case of haplotypes II-IV. **B** Prediction of the fragment size obtained by Hinf I restriction. **C** Predicted banding pattern for haplotype I and haplotypes II-IV. **D** restriction patterns obtained after Hinf I digestion of *trnH-psbA IGS* amplicons in different accessions of *Ocimum*. Sample 1 and 2 are two commercial samples declaring to contain ‘Rama’, ‘Krishna’, and ‘Vana’ Tulsi.

If the type of ‘Tulsi’ depends on genetic factors rather than on differences in light quality or developmental state, DNA-based assays to discriminate ‘Vana Tulsi’ from ‘Rama Tulsi’ and ‘Krishna Tulsi’ should target informative differences in the haplotype. We have used this idea to develop a simple assay making use of a specific single-nucleotide polymorphism in the *trnH-psbA* intergenic spacer predicted to cause a differential restriction site for HinfI (**Fig 6A**). As a result, the *trnH-psbA IGS* amplicon should not be cut for members of the haplotype I, while members of the other haplotypes would become restricted (**Fig 6B**). Upon treatment of the *trnH-psbA IGS* amplicon, one would get for specimens from haplotype I a single band of 420 bp, while for specimens from the other haplotypes, there would be two smaller fragments of 236 bp and 188 bp (**Fig 6C**). We verified this prediction restricting the *trnH-psbA IGS* amplicon from *O*. *basilicum*, *O*. *americanum*, and *O*. x *africanum* as representatives of haplotype I and did not see a breakdown of the 420-bp band (**Fig 6D**), while for *O*. *tenuiflorum*, *O*. *gratissimum*, and *O*. *tenuiflorum* a double band at around 200 bp was seen. As to be expected, restriction of Vana Tulsi yielded only the full-length band at 420 bp. Two commercial samples declared to contain ‘Rama’, ‘Krishna’, and ‘Vana Tulsi’ were assayed by the same approach (**Fig 6D**, *samples 1 and 2*): in both cases, the full-length band as well as the restriction fragments were found, indicative of a mixed sample containing both haplotype 1 and haplotype 2. Interestingly, sample 2 was declared to contain ‘Rama’ and ‘Krishna Tulsi’, specified as *O*. *sanctum*, and ‘Vana Tulsi’, specified as *O*. *gratissimum*, i.e. only genotypes that should belong to haplotype 2. Since non-foliar material from other plant material (cinnamon, ginger, pepper, cardamom) had been separated out prior to PCR, the observed fingerprint indicates that the ‘Vana Tulsi’ in the sample (if present) belongs to haplotype I.

## Discussion

### Vernacular types of Tulsi in light of the biological species concept

In the current work, we can show that plastidic barcoding markers can identify different haplotypes within the genus *Ocimum*: Haplotype II (*O*. *tenuiflorum*) clearly differs from haplotype I (*O*. *basilicum*, *O*. *americanum*, *O*. *x africanum*, and *O*. *kilimandscharicum*). Rama Tulsi and Krishna Tulsi belong to the *tenuiflorum* haplotype, while the third type, Vana Tulsi, belongs to haplotype I, although it cannot be assigned to any of the four recognised species in this cluster. This genetic difference is mirrored by differences in the profiles of secondary compounds. Developmental state and light quality can modulate the amplitude to which the chemical profile becomes manifest, possibly linked with different, light-quality dependent responses in the development of oil glands. Nevertheless, the profile itself seems to be relatively hard-wired, as seen by the robust difference between Vana Tulsi and the other two (Rama, Krishna) types of Tulsi. Our findings are consistent with a previous study [25], where ‘Ram Tulsi’ and ‘Krishna Tulsi’ diverged clearly from *O*. *basilicum*, *O*. *americanum*, *O*. *citriodorum* (a synonym of *O*. *x africanum*), and *O*. *kilimandscharicum*, although this conclusion was based on only one sequence per accession.

Vernacular nomenclature is based on differences in human use of the respective plants [2], whereas scientific taxonomy strives to create a nomenclature that reflects “natural” relationships among plants. While the classical Linnean concept of species as morphologically distinct groups of individuals has been mostly replaced by concepts in which genetic relationship and gene flow are used to define a species, the debate about what species actually are and how we can delineate them is ongoing (for a comprehensive discussion of current species concepts see [26]). In the context of “biological species” that are defined by genetic barriers, hybridisation of species, i.e. the successful breakage of these barriers, must represent the exception to the rule. However, it becomes increasingly clear that hybridisation is a powerful driving force for speciation reviewed in [27]. This is particularly relevant for plants, where genetic barriers are much more permeable than in animals. The Lamiaceae, with an estimate of some 7000 species, have so far remained recalcitrant to a consistent taxonomy—Lindley’s *bonmot* of the “*state of confusion*, *that has been gradually increasing since the days of Linnaeus*, *until it has become the disgrace of Botany*” [28] has basically remained valid. One reason may be the fact that species barriers are maintained here mainly by differences in flower morphology that provide a certain (but by no means a secure) channelling of gene flow. A classic example is the role of the pollination lever in the sympatric speciation of Central European *Salvia* [29]. Changes in the geometry of sexual organs leading to hybridisation are a likely event and are expected to generate a more or less reticulate field of different “species”. In this context, the diffuse clustering of four *Ocimum* species into haplotype I is what would be expected, while the fairly robust delineation of *O*. *tenuiflorum* into a separate haplotype is a phenomenon that demands further explanation.

Plastidic markers are generally thought to be inherited exclusively through the maternal germ line, based on different mechanisms such as mitotic exclusion of plastids or plastid degeneration in the sperm cells [30]. However, around 20% of tested Angiosperms show biparental inheritance of plastids based on different mechanisms [31]. Whether biparental inheritance is active in *Ocimum* is not known. It cannot be excluded, since one of the showcases where plastidic biparental inheritance has been demonstrated is a different member of the Lamiaceae, *Nepeta cataria* [32]. However, without further evidence, the claim to demonstrate the hybrid nature of *O*. *citriodorum* (a synonym for *O*. *x africanum*) using plastidic markers [25] seems inappropriate. Thus, the tight separation of the *tenuiflorum* haplotype from the other *Ocimum* species *per se* cannot rule out that pollen from *tenuiflorum* can fertilise flowers from haplotype I, nor that haplotype I pollen can fertilise a *tenuiflorum* flower. (These events would not leave any trace in the plastidic genome in case of maternal plastid inheritance). However, we can state that the distinct/discrete haplotype differences are found in all four tested loci of the chloroplast genome, indicative of a relatively long history of divergence, while the divergence of the different “species” within haplotype I seems to be much shorter (or not even at a stage, where it has become irreversible).

However, there is evidence for a different mechanism of reproductive isolation: A careful study based on karyotyping, flow-cytometric determination of DNA content, and nuclear markers [33] support a scenario, where *O*. *basilicum* are tetraploids deriving from an ancestor with 12 chromosomes, while *O*. *americanum* and *O*. *x africanum* are hexaploids. *O*. *x africanum* can produce viable offspring with some accessions of *O*. *basilicum* indicative of a still incomplete genetic barrier [34]. In contrast, *O*. *tenuiflorum*, has a much smaller genome which seems to derive from a hypopolyploid event, where the basal number of chromosomes had been reduced to 9. This would provide a straightforward explanation for the very tight genetic divergence of haplotype II (*O*. *tenuiflorum*), in contrast to the rather promiscuous pattern seen between the species forming haplotype I. ‘Vana Tulsi’, while clearly clustering with haplotype I, nevertheless differs from any of the tested species in this cluster and from its morphology rather resembles the accessions of haplotypes II and III, indicative of a certain degree of reproductive isolation–it might therefore be promising to investigate this type of Tulsi by cytogenetics to search for marks of incipient speciation, for instance in consequence of a hybridisation event. It is recognised that the plastidic markers used in the current study are not suited to detect hybridisations. Nuclear barcoding markers such as the internally transcribed sequences ITS1 and ITS2 might allow further resolution. Results from our previous study [35] indicate that these markers allow further differentiation within haplotype I, and we are currently including additional accessions to obtain more solid data in support of this hypothesis.

### Steps towards molecular authentication of different Tulsi types

In the current work, we have explored the potential of barcodes for authentication of ambiguous plant material for the sake of consumer safety and product transparency. For commercial application, discriminative power is not sufficient as criterion. Robust amplification success or accessibility for sequencing are further criteria that must be met. In our hands, two of the markers, *rbcL* and *trnH-psbA*, show the best potential for commercial application. While *rbcL* was the marker which was most easily amplified (for instance, if compared to *matK*), its relatively high degree of conservation represents a certain drawback. Therefore, the *trnH-psbA* barcode seems to provide a good compromise between robust amplification and discriminative power. This is consistent with the experience of others, and in fact, the introduction of this marker into the British Pharmacopoeia to authenticate *O*. *tenuiflorum* represents the first example of a DNA barcode as a trait in industrial quality assurance [36].

Despite the prospects for industrial applications, there is still a long way to go until molecular markers can be used routinely–the regulatory and technical issues have been comprehensively reviewed in [37]. For instance, many of the traded products contain mixtures of different plant species, parallel processing of a large number of samples by PCR poses the challenge that even minute contamination can be amplified creating false positive results and DNA quality in what are often processed samples may become limiting. A further drawback of authentication by barcoding is the need to generate and analyse the sequences before a conclusion on the identity of the sample can be made. This is time consuming and requires expertise in handling sequence information, which conflicts with the requirements of practical use, where large number of samples may have to be checked in a short time to decide whether they can be released for trading. Fingerprinting methods, such as random amplified polymorphic DNA (using arbitrary primer pairs to generate a banding pattern) are convenient at first sight, because they do not require any working hypothesis. However, the reliability of these markers for authentication is limited as shown by systematic validation testing in the Brassicaceae [38]. The reason is mainly that the relatively low annealing temperatures for these arbitrary primers can generate non-specific amplification products. Also, bands of equal size do not necessarily originate from the same template but are treated as if they were. Alternative markers, such as inter-simple sequence repeats (ISSR), allow for a better resolution as compared to the plastidic barcodes [39]. However, for the same reasons as for RAPD (low annealing temperatures, ambiguous assignment of banding patterns), the ISSR approach can have reproducibility issues [40], which may be tolerable in a taxonomic study, but strongly conflicts with the needs of consumer protection.

Assays to authenticate commercial samples must meet three central requirements: they have to be fast, they have to be easy, and they have to be reliable. Our approach, targeting a restriction polymorphism of the *trnH-psbA* marker, meets these requirements. We exemplarily applied this strategy to two commercial samples declared to contain mixtures of ‘Rama’, ‘Krishna’, and ‘Vana Tulsi’, (which was declared as *O*. *gratissimum*) and could show that the declaration of ‘Vana Tulsi’ as *O*. *gratissimum* was wrong.

A similar restriction-based strategy (using the *rbcL* marker) has been used successfully to authenticate two species that are used in the traditional medicine of the Australian Aborigines but now traded under the same vernacular name, Lemon Myrtle [41]. Alternatively, the sequence differences can be used to design specific destabilised oligonucleotide primers that will only bind one target, but not the other and can be added to the conventional barcoding primers. This so-called Amplification Refractory Mutation System (ARMS) will thus yield a diagnostic side band in addition to the full-length amplicon. Using this strategy, it is possible to discriminate the Moldavian Dragonhead (*Dracocephalum moldavica*) from Cat Mint (*Nepeta cataria* L.) and Common Melissa (*Melissa officinalis* L.), species that are often used as surrogates [42]. Likewise, adulteration of Medicinal Bamboo Tea by a species of Chinese Carnation in Germany could be detected using the ARMS approach [5]. Which of these alternative strategies can be applied, depends on the particular sequence motifs. Compared to the restriction-based assay, the ARMS approach is faster. Moreover, the full-length amplicon in addition to the diagnostic side band provides an inbuilt control for the success of the PCR [42].

### Outlook

Making use of sequence differences in the barcoding marker *trnH-psbA*, we were able to discriminate different types of the medicinal plant Tulsi and to clarify the phylogenetic relationship of Vana Tulsi (haplotype I) versus Rama and Krishna Tulsi (haplotype II). Further, we developed a simple and rapid assay to discriminate these types of Tulsi based on a differential restriction site in this marker. Although this assay can contribute to improved transparency, because it highlights differences which align to vernacular designations of commercial products, further progress in discriminative resolution would be desirable. Given the impact of genome remodelling during the evolution of *Ocimum* [33], duplications in gene families would be one of the options–a principle exploited in Tubulin-Based Polymorphism, a strategy with high resolutive power [43]. For instance, the practice to commercialise Tulsi in complex mixtures with other Ayurvedic plants clearly illustrates the limitation even of *trnH-psbA* as trait-independent marker. For practical applications, it is the chemical specificities that matter. Our observation that the compound profiles seem to be based on genetic differences, while environmental conditions (light quality) and development instead modulate the degree to which these profiles become manifest, provides a good starting point to develop trait-related markers that would discriminate specific chemotypes of *Ocimum*. This would also allow authentication of target species in a mixed sample without the need for demanding approaches such as high-throughput genomics of commercial samples.

## Supporting information

S1 Fig Molecular relationships inferred from the RubisCo large subunit (*rbcL*) marker using the data set shown in Fig 2 and two alternative algorithms (neighbour joining versus maximum likelihood). (TIF)

S2 Fig Molecular relationships inferred from the *matK* marker. **A** Evolutionary relationship of 64 sequences for the *matK* marker from *Ocimum*, along with one sequence from *Nepeta cataria* and *Hanceola sinensis*, respectively, used as outgroups inferred using the Neighbor-Joining algorithm. Bootstrap values are derived from 1000 replicates. O bas *O*. *basilicum*, O americ *O*. *americanum*, O x afric *O*. x *africanum*, O kilim *O*. *kilimandscharicum*, O campech *O*. *campechianum*, O carnos *O*. *carnosum*, O tenuifl *O*. *tenuiflorum*, O fil *O*. *filamentosum*, O grat *O*. *gratissimum*. GenBank accession numbers are shown with each accession, circles represent sequences that had been isolated in the course of the current study, red circle represents the sequence for ‘Vana Tulsi’ that clusters outside of *O*. *tenuiflorum*. Blue arrows indicate two accessions that have been reported as type ‘Krishna Tulsi’ (both as *O*. *tenuiflorum*), green arrows indicate two accessions that have been reported as ‘Vana Tulsi’. Roman numbers represent the four main haplotypes, letters subgroups revealed by the *matK* marker. **B** Signatures of the haplotypes defined by the *matK* marker, numbers indicate the nucleotide position in the alignment (**S2 Appendix**). Support is defined as the number of sequences deposited in GenBank that show this signature. Note that the sequence of ‘Vana Tulsi’ falls into cluster I comprising *O*. *basilicum*, *O*. *americanum*, *O*. x *africanum*, and *O*. *kilimandscharicum*.
(TIF)

S3 Fig Molecular relationships inferred from the *trnL-F* spacer. **A** Evolutionary relationship of 37 sequences for the *trnL-F* spacer from *Ocimum*, along with one sequence from *Nepeta cataria* and *Hanceola sinensis*, respectively, used as outgroups inferred using the Neighbour-Joining algorithm. Bootstrap values are derived from 1000 replicates. O bas *O*. *basilicum*, O americ *O*. *americanum*, O x afric *O*. x *africanum*, O tenuifl *O*. *tenuiflorum*, O fil *O*. *filamentosum*, O grat *O*. *gratissimum (*grat var. *gratissimum*, suave var. *suave*, macr var. *macrophyllum)*. O ser *O*. *serratum*, O lab *O*. *labiatum*. GenBank accession numbers are shown with each accession, circles represent sequences that had been isolated in the course of the current study, red circle represents the sequence for ‘Vana Tulsi’ that clusters outside of *O*. *tenuiflorum*. Blue arrow indicates our validated accession for ‘Krishna Tulsi’, green arrow indicates our validated accession for ‘Vana Tulsi’. Roman numbers represent the four main haplotypes, letters subgroups revealed by the *trnL-F* marker. **B** Signatures of the haplotypes defined by the *trnL-F* marker, numbers indicate the nucleotide position in the alignment (**S3 Appendix**). Support is defined as the number of sequences deposited in GenBank that show this signature. Note that the sequence of ‘Vana Tulsi’ clusters with *O*. *basilicum* and not with the other accessions of *O*. *tenuiflorum*. Green square indicates the only position in the alignment, where ‘Vana Tulsi’ shares a signature with *O*. *tenuiflorum* and not with *O*. *basilicum*. Gaps are indicated by 0 (absence of nucleotides) and 1 (presence of nucleotides), respectively.
(TIF)

S4 Fig Molecular relationships inferred from the *trnH-psbA* intergenic spacer. **A** Evolutionary relationship of 79 sequences for the *trnH-psbA* intergenic spacer from *Ocimum*, along with one sequence from *Nepeta cataria* and *Hanceola sinensis*, respectively, used as outgroups inferred using the Neighbor-Joining algorithm. Bootstrap values are derived from 1000 replicates. O bas *O*. *basilicum*, O americ *O*. *americanum*, O x afric *O*. x *africanum*, O tenuifl *O*. *tenuiflorum*, O fil *O*. *filamentosum*, O grat *O*. *gratissimum (*suave var. *suave)*. O campech *O*. *campechianum*, O selloi *O*. *selloi*. GenBank accession numbers are shown with each accession, circles represent sequences that had been isolated in the course of the current study, red circle represents the sequence for ‘Vana Tulsi’ that clusters outside of *O*. *tenuiflorum*. Blue arrow indicates our validated accession for ‘Krishna Tulsi’, green arrow indicates our validated accession for ‘Vana Tulsi’. Roman numbers represent the four main haplotypes, letters subgroups revealed by the *trnL-F* marker. **B** Signatures of the haplotypes defined by the *trnH-psbA* marker, numbers indicate the nucleotide position in the alignment (**S4 Appendix**). Support is defined as the number of sequences deposited in GenBank that show this signature. Note that the sequence of ‘Vana Tulsi’ clusters with *O*. *basilicum* and not with the other accessions of *O*. *tenuiflorum*. Gaps are indicated by 0 (absence of nucleotides) and 1 (presence of nucleotides), respectively.
(TIF)

S5 Fig Flavonoid profiling of Rama Tulsi, Krishna Tulsi, and Vana Tulsi followed through different developmental stages raised side by side in the greenhouse under white light over up six months as probed by HP-TLC. As reference standards, rutin and hyperoside, were used. Mature seeds, young plants (raised for 2 months), and adult plants (raised for 6 months) are shown along with commercial samples of the same genotypes that were grown in the field in India.
(TIF)

S1 Appendix Alignment of 65 sequences for the *rbcL* marker from *Ocimum*, along with one sequence from *Nepeta cataria* and *Hanceola sinensis*, used as outgroup, generated by the ClustalW algorithm. (TXT)

S2 Appendix Alignment of 64 sequences for the *matK* marker from *Ocimum*, along with one sequence from *Nepeta cataria* and *Hanceola sinensis*, used as outgroup, generated by the ClustalW algorithm. (TXT)

S3 Appendix Alignment of 37 sequences for the *trnL-F* intergenic spacer from *Ocimum*, along with one sequence from *Nepeta cataria* and *Hanceola sinensis*, used as outgroup, generated by the ClustalW algorithm. (TXT)

S4 Appendix Alignment of 79 sequences for the *trnH-psbA* intergenic spacer from *Ocimum*, along with one sequence from *Nepeta cataria* and *Hanceola sinensis*, used as outgroup, generated by the ClustalW algorithm. (TXT)
