# Supplementary figures and images for: Product authenticity versus globalisation—The Tulsi case
Source: PLoS One. 2018 Nov 26;13(11):e0207763. doi: 10.1371/journal.pone.0207763 (PMC6261265; doi:10.1371/journal.pone.0207763)

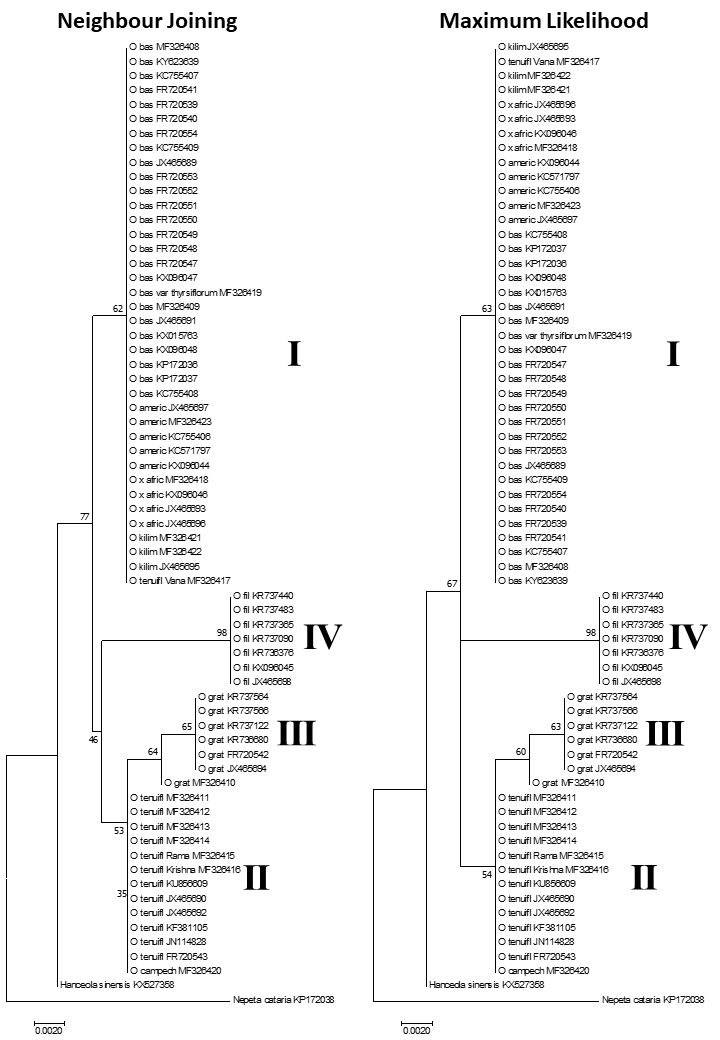

Supplement: S1 Fig — (TIF) [file pone.0207763.s001.TIF]

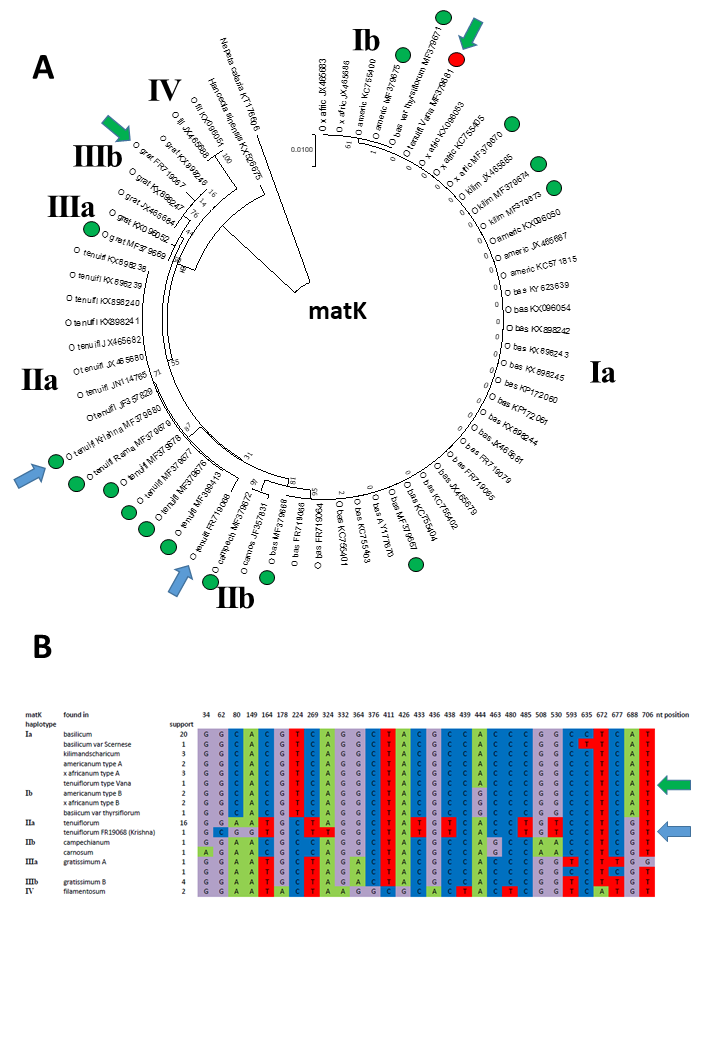

Supplement: S2 Fig — A Evolutionary relationship of 64 sequences for the matK marker from Ocimum, along with one sequence from Nepeta cataria and Hanceola sinensis, respectively, used as outgroups inferred using the Neighbor-Joining algorithm. Bootstrap values are derived from 1000 replicates. O bas O. basilicum, O americ O. americanum, O x afric O. x africanum, O kilim O. kilimandscharicum, O campech O. campechianum, O carnos O. carnosum, O tenuifl O. tenuiflorum, O fil O. filamentosum, O grat O. gratissimum. GenBank accession numbers are shown with each accession, circles represent sequences that had been isolated in the course of the current study, red circle represents the sequence for ‘Vana Tulsi’ that clusters outside of O. tenuiflorum. Blue arrows indicate two accessions that have been reported as type ‘Krishna Tulsi’ (both as O. tenuiflorum), green arrows indicate two accessions that have been reported as ‘Vana Tulsi’. Roman numbers represent the four main haplotypes, letters subgroups revealed by the matK marker. B Signatures of the haplotypes defined by the matK marker, numbers indicate the nucleotide position in the alignment (S2 Appendix). Support is defined as the number of sequences deposited in GenBank that show this signature. Note that the sequence of ‘Vana Tulsi’ falls into cluster I comprising O. basilicum, O. americanum, O. x africanum, and O. kilimandscharicum. (TIF) [file pone.0207763.s002.TIF]

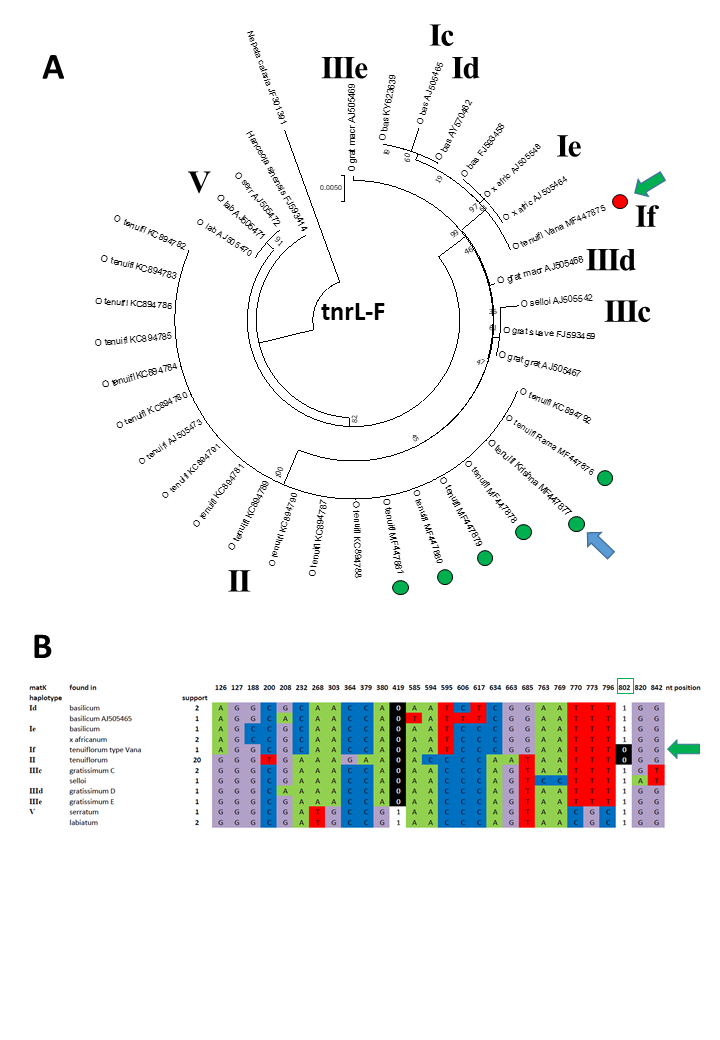

Supplement: S3 Fig — A Evolutionary relationship of 37 sequences for the trnL-F spacer from Ocimum, along with one sequence from Nepeta cataria and Hanceola sinensis, respectively, used as outgroups inferred using the Neighbour-Joining algorithm. Bootstrap values are derived from 1000 replicates. O bas O. basilicum, O americ O. americanum, O x afric O. x africanum, O tenuifl O. tenuiflorum, O fil O. filamentosum, O grat O. gratissimum (grat var. gratissimum, suave var. suave, macr var. macrophyllum). O ser O. serratum, O lab O. labiatum. GenBank accession numbers are shown with each accession, circles represent sequences that had been isolated in the course of the current study, red circle represents the sequence for ‘Vana Tulsi’ that clusters outside of O. tenuiflorum. Blue arrow indicates our validated accession for ‘Krishna Tulsi’, green arrow indicates our validated accession for ‘Vana Tulsi’. Roman numbers represent the four main haplotypes, letters subgroups revealed by the trnL-F marker. B Signatures of the haplotypes defined by the trnL-F marker, numbers indicate the nucleotide position in the alignment (S3 Appendix). Support is defined as the number of sequences deposited in GenBank that show this signature. Note that the sequence of ‘Vana Tulsi’ clusters with O. basilicum and not with the other accessions of O. tenuiflorum. Green square indicates the only position in the alignment, where ‘Vana Tulsi’ shares a signature with O. tenuiflorum and not with O. basilicum. Gaps are indicated by 0 (absence of nucleotides) and 1 (presence of nucleotides), respectively. (TIF) [file pone.0207763.s003.TIF]

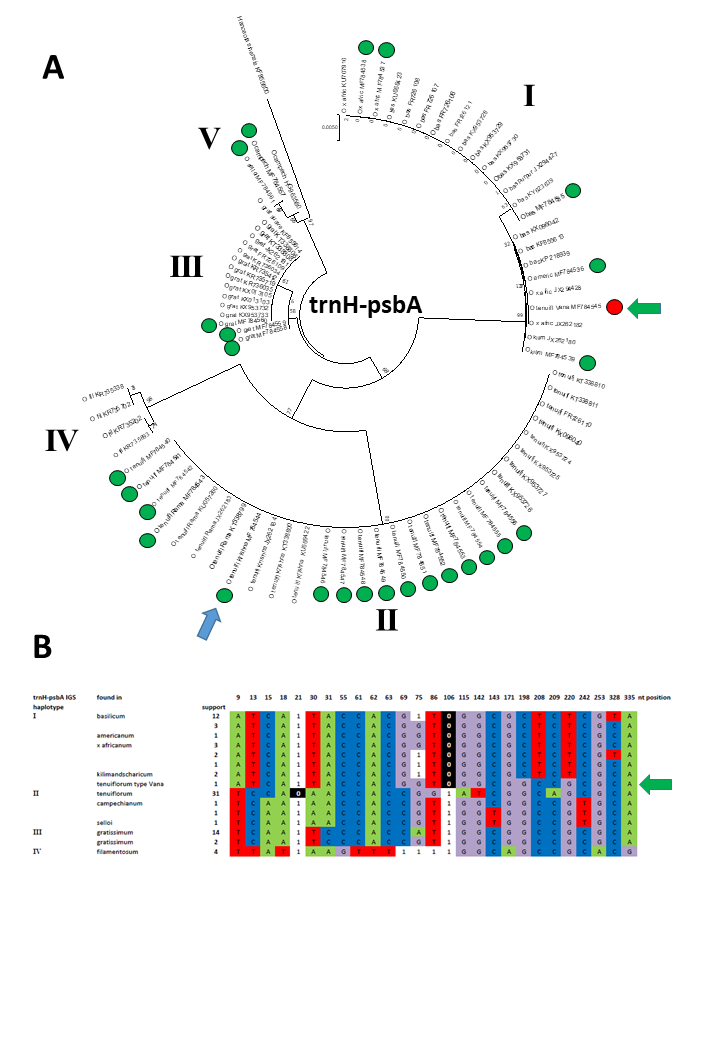

Supplement: S4 Fig — A Evolutionary relationship of 79 sequences for the trnH-psbA intergenic spacer from Ocimum, along with one sequence from Nepeta cataria and Hanceola sinensis, respectively, used as outgroups inferred using the Neighbor-Joining algorithm. Bootstrap values are derived from 1000 replicates. O bas O. basilicum, O americ O. americanum, O x afric O. x africanum, O tenuifl O. tenuiflorum, O fil O. filamentosum, O grat O. gratissimum (suave var. suave). O campech O. campechianum, O selloi O. selloi. GenBank accession numbers are shown with each accession, circles represent sequences that had been isolated in the course of the current study, red circle represents the sequence for ‘Vana Tulsi’ that clusters outside of O. tenuiflorum. Blue arrow indicates our validated accession for ‘Krishna Tulsi’, green arrow indicates our validated accession for ‘Vana Tulsi’. Roman numbers represent the four main haplotypes, letters subgroups revealed by the trnL-F marker. B Signatures of the haplotypes defined by the trnH-psbA marker, numbers indicate the nucleotide position in the alignment (S4 Appendix). Support is defined as the number of sequences deposited in GenBank that show this signature. Note that the sequence of ‘Vana Tulsi’ clusters with O. basilicum and not with the other accessions of O. tenuiflorum. Gaps are indicated by 0 (absence of nucleotides) and 1 (presence of nucleotides), respectively. (TIF) [file pone.0207763.s004.TIF]

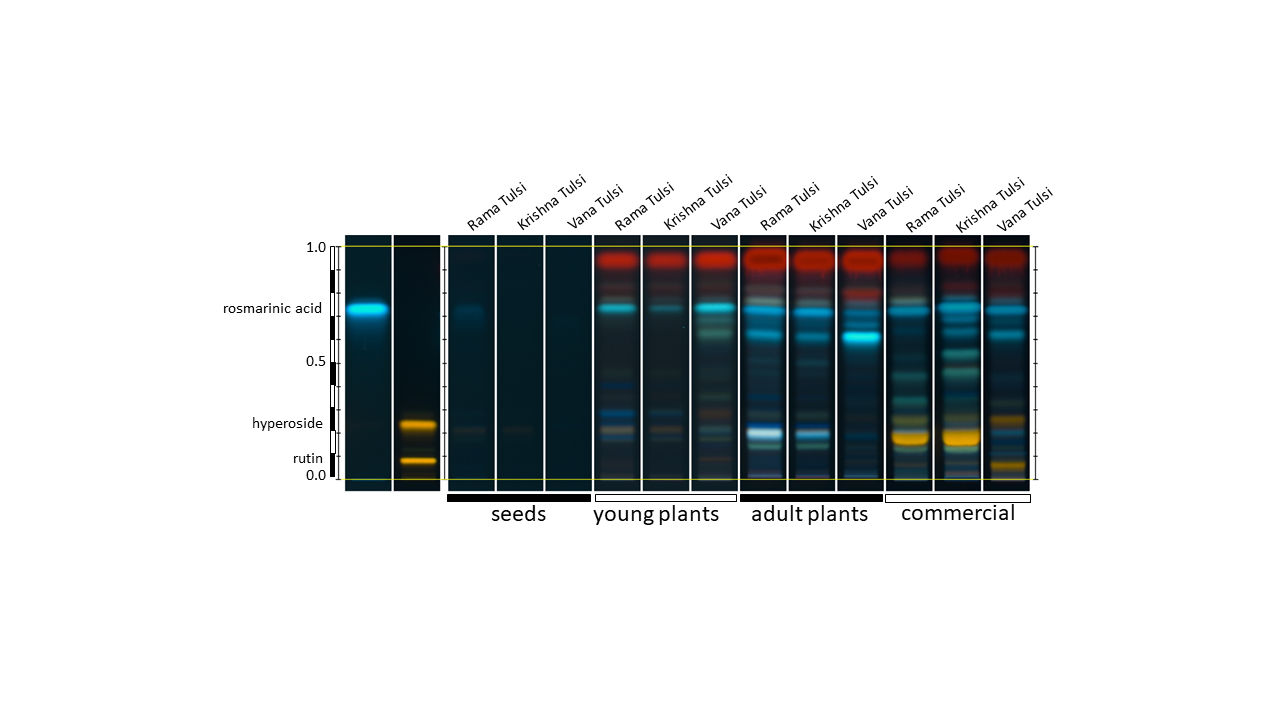

Supplement: S5 Fig — As reference standards, rutin and hyperoside, were used. Mature seeds, young plants (raised for 2 months), and adult plants (raised for 6 months) are shown along with commercial samples of the same genotypes that were grown in the field in India. (TIF) [file pone.0207763.s005.TIF]
